# Linking COVID-19, monocyte activation and sepsis: MDW, a novel biomarker from cytometry

**DOI:** 10.1016/j.ebiom.2021.103754

**Published:** 2021-12-15

**Authors:** Giovanni Riva, Vincenzo Nasillo, Mario Luppi, Enrico Tagliafico, Tommaso Trenti

**Affiliations:** a Diagnostic Hematology and Clinical Genomics Laboratory, Department of Laboratory Medicine and Pathology, AUSL/AOU Modena, Policlinico, Via del Pozzo 71, Modena 41124, Italy; b Section of Hematology, Department of Medical and Surgical Sciences, University of Modena and Reggio Emilia, AOU Modena, Policlinico, Via del Pozzo 71, Modena 41124, Italy

Circulating monocytes, recruited from the bone marrow to injured organs upon infection or tissue damage, stay at the complex intersection of immune defence, systemic inflammation and tissue repair. Indeed, monocytes and monocyte-derived macrophages are key regulators of both innate and adaptive cellular immunity, as well as humoral inflammation, complement cascade and plasma coagulation. Upon activation, these cells undergo structural and functional modifications, enabling a broad set of immunomodulatory activities, mainly exerted by either proinflammatory or inhibitory cytokines, chemokines, and other inflammatory mediators, as well as by cell-to-cell interactions and phagocytosis. As supported by several lines of evidence, monocyte/macrophage dysregulation may represent the cornerstone of the systemic hyper-inflammatory response (cytokine storm), coagulopathy and lung damage (respiratory failure), characterizing the severe form of COVID-19.^1^

Monocyte Distribution Width (MDW), an emerging cytometric parameter, automatically calculated within complete blood count by novel haematology analyzers, is linked to volume modifications occurring in the whole monocyte population upon massive immune activation. Consistent with this, MDW was originally described as an ‘early sepsis indicator’ in the Emergency Department (ED) and Intensive Care Unit (ICU), and, recently, different reports have shown some promising MDW applications also in COVID-19 patients. Together, these works well evidenced that MDW is significantly higher in COVID-19 than in non-COVID-19 cases, thus suggesting that upfront MDW measurement, while awaiting SARS-CoV-2 molecular results, could allow a prompt differential diagnosis in the emergency setting.^2^ Intriguingly, by providing a conceptual link between the diagnosis of sepsis and COVID-19, MDW findings can further support the notion that severe COVID-19 may constitute a (new) kind of viral sepsis.^3^ Moreover, recent pilot studies have disclosed that MDW testing may also be exploited for prognostic monitoring in hospitalized COVID-19 patients, either alone,^4^ or in combination with Neutrophil-to-Lymphocyte Ratio (NLR).^5^

In this view, by considering both the biological rationale and clinical data reported so far, MDW can represent a useful haematological (cell-based) parameter, related to monocyte activation typically occurring in life-threatening (dis)inflammatory conditions, such as sepsis and COVID-19. In particular, MDW could be relevant as a prognostic factor in the management of COVID-19 patients (Figure 1). Further investigations are warranted to define optimal MDW thresholds, potentially improving the continuous risk assessment in COVID-19 patients, at different clinical stages. In perspective, additional studies involving large cohorts are needed to shed more light on the prognostic role of MDW in this context, possibly in combination with other cellular and humoral biomarkers, aiming to identify predictive tools for helping the therapeutic decision-making in patients with SARS-CoV-2 infection.

**Figure 1:**
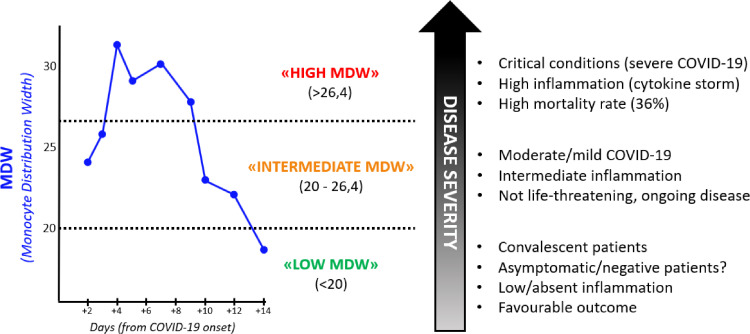
A working model for MDW use in COVID-19 patients. The novel haematological parameter MDW, already described as early sepsis biomarker in ED and ICU settings, can well correlate with COVID-19-related systemic inflammation, disease severity and outcome.^4^ Here, three risk groups (*low, intermediate* and *high MDW*) are identified on the basis of reported MDW cutoffs.^2^^,^^4^^,^^5^

## Contributors

Conception and writing: GR and VN. Critical revision: ML, ET and TT. All authors approved the final version of the manuscript.

## Declaration of interest

The authors declare no competing interests.
